# Resolving the Ethical Tension Between Creating a Civil Environment and Facilitating Free Expression Online: Comment Reordering as an Alternative to Comment Moderation

**DOI:** 10.1007/s10551-023-05450-9

**Published:** 2023-06-14

**Authors:** Dena Yadin, Inbal Yahav, Lior Zalmanson, Nira Munichor

**Affiliations:** 1 D’Amore-McKim School of Business, Northeastern University, Boston, MA 02115 USA; 2 Coller School of Management, Tel Aviv University, 6997801 Tel Aviv, Israel; 3 Graduate School of Business Administration, Bar Ilan University, 5290002 Ramat-Gan, Israel

**Keywords:** User-generated-content, Online incivility, Content moderation

## Abstract

Incivility in online commenting sections can create a hostile environment and result in the silencing of vulnerable voices. Accordingly, content websites and social media platforms have an ethical responsibility—one that aligns with their strategic interests—to minimize users' exposure to uncivil content. To this end, platforms invest great effort and budget in automatic and manual filtering mechanisms. Yet, these efforts create a competing ethical quandary, as they often come at the expense of free expression, particularly in cases where comments do not explicitly infringe on stated guidelines but might nevertheless be interpreted as offensive. In this paper, we examine an alternative moderation approach, based on comment reordering as opposed to deletion of uncivil comments. Specifically, we show that exposure to uncivil (vs. civil) comments located in the head or at the bottom of a list of comments increases subsequent commenters' likelihood of posting uncivil comments themselves. Exposure to uncivil comments in the middle of a list, however, does not significantly enhance commenters' likelihood of commenting uncivilly. These results offer new theoretical insight into how incivility is transferred between users in online environments. Our results also suggest a straightforward technological solution for mitigating online incivility, which is more ethical and practical than current industry standards. This involves placing civil comments at the beginning and end of the comment thread, with uncivil comments located in the middle.

## Introduction

With the rise of social media, user comments and other forms of user-generated content (UGC) have begun to occupy an increasingly prominent role on content websites (Oestreicher-Singer & Zalmanson, 2013). This shift has created clear benefits for users and for website owners, as well as for society at large. Specifically, active comment sections enable users to interact with one another (Winer, 2009), create and disseminate their own content, express their thoughts, and fulfill personal goals, such as the need for recognition and meaning, while potentially enhancing the experience of fellow users (Burtch et al., 2018; Khansa et al., 2015). From website owners’ perspective, the surge in user contributions has provided a stream of free content that triggers engaging discussions, which can increase users’ time on the website, their retention rates (Huang et al., 2019), and even their willingness to pay for website services (e.g., Oestreicher-Singer & Zalmanson, 2013). On a societal level, the availability of platforms for user feedback echoes an overall rise in free expression (Cohen-Almagor, 2012), which has allowed for acknowledgment of marginalized voices (Drake et al., 2000) and contributed toward the building of grassroots social movements such as MeToo and Black Lives Matter. Moreover, the content that users generate can contribute to the public good by externalizing people’s knowledge and experience (Drake et al., 2000; Ransbotham et al., 2016).

Alongside the benefits of comment sections, however, new challenges and vulnerabilities have arisen (Cohen-Almagor, 2012; Drake et al., 2000; Rauf, 2021). One particularly concerning phenomenon is the emergence of online incivility—a term that encompasses numerous behaviors such as flaming (aggressive online arguments), posting of offensive content, trolling (posting inflammatory comments with the primary goal of triggering emotional responses), bullying, harassing fellow users, and intentionally derailing conversations (Chan et al., 2019). Online incivility can hinder the website experience by creating a hostile environment for content sharing and the exchange of ideas (Cohen-Almagor, 2012; Van Laer, 2014). And the phenomenon is widespread: A study by Coe et al. (2014) indicates that one in five online comments is uncivil. Moreover, a recent report from the Pew Research Center (Vogels, 2021) suggests that online incivility is becoming more severe: For example, a comparison of survey data collected at different points in time showed that larger percentages of people reported experiencing severe harassment in 2020 than in 2017 (25% vs. 18%, respectively)—though the percentage of people who experienced any form of online incivility remained more or less the same (around 41%). In fact, recent studies have suggested that the level of online harassment and incivility is growing to a point where it may be detrimental to the future of the internet (Stein, 2016) as well as to local and global political processes. Incivility may threaten and silence marginalized voices, polarize society, and ultimately jeopardize democracy (Gervais, 2015).

Websites and online platforms, which manage their users’ overall experience (Cohen-Almagor, 2012), are ethically responsible for ensuring that their users do not suffer from hostility and harassment. Moreover, failure to create a safe and civil environment not only represents a moral concern but also creates significant business vulnerabilities. For example, incivility negatively affects users’ perceptions of the quality of a platform’s content (Prochazka et al., 2018). Excessive incivility may also cause users to defect (Van Laer, 2014) and spread negative word-of-mouth about the website (Bacile et al., 2018). It may also expose websites to lawsuits from individuals and national authorities.^1^

In light of these concerns, many websites have content moderation practices in place to mitigate incivility (Cohen-Almagor, 2012; Van Laer, 2014). These practices commonly rely on manual (Chen et al., 2011) or semi-automated procedures (O’Brien, 2019; Riedl et al., 2020) that identify posted content as uncivil and subsequently remove it from the website. These practices are far from ideal: First, they are extremely expensive and time-consuming (Gonçalves et al., 2021; Riedl et al., 2020). Second, moderation practices might not be totally effective, as it is common for unusual or subtle forms of incivility, which are potentially difficult to detect, to remain in the thread (Coe et al., 2014). Third, and most importantly for our context, they introduce a new ethical quandary that is in tension with website owners’ responsibility to prevent incivility: Removing comments may compromise users’ freedom of speech, a value that has become intertwined with the ideal notion of the internet as a “public sphere” that is fundamentally democratic and egalitarian (see further discussion below) (Habermas, 1989; Jhaver et al., 2019). This concern is intensified by the fact that content moderation, particularly when done manually, can be highly subjective, which may result in biases with regard to which content is removed versus allowed to remain (O’Brien, 2019; Riedl et al., 2020).

The current study proposes an applicable approach to resolving this tension, one that enables potentially uncivil content to be retained on a website, while mitigating the harm it might cause. Specifically, our approach relies on adjustment of the layout of the comment section. We begin with the premise that some of the damage caused by incivility stems from the fact that, in certain circumstances, incivility can be contagious, such that one uncivil comment inspires other commenters to post uncivil content of their own (e.g., Suh et al., 2018). Drawing from a theory called the serial position effect, we propose that the extent to which incivility is contagious depends on the position of the uncivil content in the list of comments, for example, at the head or tail of the list versus in the middle. This proposition implies that it might be possible to curb the spread of incivility in comment sections without deleting the comments themselves—by ensuring that uncivil comments are positioned in spots where they are unlikely to trigger uncivil behavior of others. Of note, rearrangement of comments is already in use in many social networks, albeit for different purposes, such as increasing the visibility of relevant, or highly liked comments. This work proposes that a similar practice can serve as an effective means of decreasing incivility.

We test our proposition in three studies. First, we analyze observational data from the comment section of a large online content website. Next, we carry out two controlled internet experiments using Facebook-like articles and comments; this setup enables us to control the serial positions of comments to which users are exposed and to analyze their effects on users’ incivility as well as on other outcomes of interest (e.g., attention and commenting likelihood). Our findings indeed reveal an effect of comment positioning on the extent to which incivility is contagious. Comments that are located at the head or tail of the comment list are significantly more likely to trigger subsequent incivility compared with uncivil comments that are placed in the middle of the list. Notably, comments located at the tail of the comment list are particularly contagious. Our findings suggest a practical solution for reducing online incivility in content website comment sections: placing civil comments at the beginning and the end of the comment thread and positioning uncivil comments in the middle. By adjusting the layout of user-generated comments we may constitute a means of mitigating incivility without significantly limiting freedom of speech and with minimal manual intervention.

## Theoretical Background

### Online Incivility: Definition and Ethical Concerns

Incivility is a challenging term to define objectively (Rossini, 2020; Su et al., 2018). What is perfectly civil to one person might strike another person as highly uncivil (Kenski et al., 2020). Nevertheless, scholars agree on common themes of incivility, such as inappropriate vocabulary and an absence of courtesy (e.g., Coe et al., 2014; Gervais, 2015; Kenski et al., 2020; Papacharissi, 2004; Rossini, 2020; Sydnor, 2018). For our purposes, we adopt Coe et al.’s (2014) definition of incivility as ‟features of discussion that convey an unnecessarily disrespectful tone toward the discussion forum, its participants, or its topics” (p. 660). This definition is appropriate for our context since it was initially formed by Coe et al. (2014) to study comments on a content website. Kenski et al. (2020) have suggested that this conceptual definition includes most of the dimensions employed in other definitions of incivility in prior research.

Until relatively recently, it was not uncommon for online platforms to avoid intervening in uncivil interactions among their users. One motivation to refrain from intervening may be the belief that incivility can increase platform popularity. Indeed, some studies of online platforms have found that incivility can raise user engagement (Brooks & Geer, 2007; Rains et al., 2017) and online participation (Borah, 2014), and make social media seem entertaining (Sydnor, 2018). Still, other studies have failed to reveal similar positive effects (Muddiman et al., 2020; Pang et al., 2016), and in recent years, prominent internet analysts and the public have expressed increasing concerns regarding the capacity of incivility to encourage subsequent negative online behaviors (Anderson et al., 2014; Chen et al., 2011; Rainie, 2017). The victims targeted by online incivility may experience anxiety, depression, and social isolation (Ransbotham et al., 2016). Online incivility may also affect those who have not been directly targeted; for example, it may lead to silencing of minority voices that are most often targeted by harassers (Ordoñez & Nekmat, 2019; Pang et al., 2016), and cause additional segments of the population to withdraw from the social internet because of the hostility of the environment, leading to further polarization of society (Anderson et al., 2014; Ransbotham et al., 2016; Van Laer, 2014).^2^

In light of these findings, users and regulators are increasingly holding content websites and UGC platforms accountable for maintaining a civil atmosphere. Nowadays, high levels of incivility can damage websites’ reputations and cause loss of advertising revenue, as well as legislative backlash. For instance, Bacile et al. (2018) found evidence that users actively assess how a firm handles uncivil interactions on its platform and form negative perceptions of firms that fail to address perpetrators of incivility—even when the users themselves are not the targets. Likewise, Meltzer (2015), who surveyed journalists regarding their perceptions of uncivil behavior online, found that most respondents expressed a belief that they or their organizations are responsible for keeping their sites civil. In line with this notion of accountability, news sites and social networks, such as Google, Facebook, and, until recently, Twitter, are experimenting with new ways to filter out or label harmful or misleading discourse (Duggan, 2014; Rainie et al., 2017; see also Suh et al., 2018). However, a recent Pew Research survey suggests that the vast majority of the public believes that social media platforms are doing only a fair job addressing these problems (Vogels, 2021).

### Approaches to Mitigating Online Incivility

In light of the substantial societal and strategic concerns associated with online incivility, a growing stream of literature is seeking to identify means of mitigating such behavior. Studies in this stream can be divided into three groups. The first focuses on understanding root causes underlying negative human behaviors that manifest online; these factors include age and gender differences (Alonzo and Aiken, 2004; Hmielowski et al., 2014; Vochocová & Rosenfeldová, 2019), the topic of discussion (Stroud et al., 2015; Su et al., 2018; Ziegele & Jost, 2020), opinion about the subject of discussion (Gervais, 2015), social learning processes, and cultural conditions (Lowry et al., 2016). Yet, it is difficult to translate the findings of these studies into concrete solutions for mitigating incivility. For example, limiting access to certain populations of users on the basis of their inherent tendency to behave uncivilly is both impractical and unethical, as it would constitute discrimination.

The second and third groups of studies focus on factors that are more under the control of website owners and decision-makers. In particular, studies included in the second group test how a platform’s interactions with its users affect users’ commenting behavior. Stroud et al. (2015), for example, showed that on platforms in which a representative responds to comments—especially if the representative is identified and well-known—users are less likely to comment uncivilly than on platforms that do not engage with users in this manner. The representative’s response style may also matter. Ziegler and Jost (2020) found that site users perceived the commenting atmosphere as more civil when a representative’s responses were factual (i.e., constructively and politely phrased) rather than sarcastic (i.e., satirically and ironically phrased). Interestingly, the factual style of the responses also positively affected participants’ willingness to participate in the discussion. These findings suggest that engagement with commenters may facilitate incivility moderation by mitigating users’ initial tendency to behave uncivilly, as well as by increasing the prevalence of civil comments (by increasing the response rate). However, for many platforms, it may be infeasible to meaningfully interact with users on a continuous basis.

The third group of studies examines how users’ commenting behavior is influenced by technological solutions related to a platform’s design. Such solutions might include automated incentives or prompts that are presented to individual users as a means of encouraging or discouraging certain types of interactions. For example, studies suggest that by offering personal incentives to commenters, such as an online social status or reward points, platforms can enhance users’ likelihood of posting comments in general (van der Does & Bos, 2021) and can also increase comment quality (Towne & Herbsleb, 2012). Another study (see Maher, 2016) suggested that prompting users about the effects of their uncivil actions can prevent them from behaving uncivilly, at least during the brief time period following the prompt. More broadly, Chan et al. (2019) noted that, by publicizing strict and clear rules regarding acceptable conduct, and by consistently enforcing them (e.g., warning and subsequently suspending users who behave inappropriately), a website may further enhance its capacity to deter uncivil behavior.

Other studies in this group focus on user identifiability as a platform design feature with the potential to affect the prevalence of uncivil behavior. Zimmerman and Ybarra (2016), for example, showed that user identifiability (vs. anonymity) can mitigate online incivility, and thus suggested identity-enforcement as a potential means of reducing incivility. Yet, additional studies raise doubts regarding the efficacy of user identifiability as a remedy for online incivility: Rösner and Krämer (2016) showed that in some online settings, the behavior of others has a more significant effect on commenting behavior than anonymity does. This finding was reinforced by a recent study by Rossini (2020), who found that on Facebook—where users’ identity is explicit and salient—users behaved more uncivilly compared with anonymous commenters on news outlets. Moreover, even if identifiability-enforcement is an effective approach to reducing incivility, it might present additional shortcomings, as requiring users to identify with their names may deter certain people from participating (Friess & Eilders, 2015).

Technological solutions for influencing users’ commenting behavior may also involve structural changes to the comment section. For example, Peacock et al. (2019) showed that by merely changing the form of a comment section from a one-column structure to a three-column structure, and by providing arguments for and against a particular issue, it is possible to increase the number of comments posted on the issue, as well as the “deliberative quality” of those comments (as reflected, e.g., in the comments’ relevance to the topic being discussed, the extent to which they are supported by evidence, and their level or flexibility and openness to multiple viewpoints).

In spite of findings regarding the potential for technological solutions to contribute positively to the quality of online discussion, large numbers of platforms continue to rely primarily on manual or semi-automated content moderation solutions (e.g., Chen et al., 2011; O’Brien, 2019; Riedl et al., 2020)—which are practically challenging to implement. Of course, an even more straightforward solution for preventing incivility would be to eliminate comment sections entirely (Chen et al., 2011; Gonçalves et al., 2021; Huang, 2016; Meltzer, 2015). Yet, denying users the opportunity to comment, or deleting comments that are deemed inappropriate, raises ethical concerns, as elaborated in the following subsection.

### The Ethical Quandary of Content Moderation in Online Settings

Commercially owned websites, as so-called private actors, are not bound by free speech laws (Cohen-Almagor, 2012; Pinkus, 2021). Thus, they are fully within their rights to eliminate user content as they see fit, particularly when this is done with the aim of providing a civil environment. Nevertheless, content elimination creates a certain ethical tension, as it hinders freedom of expression—a value that many people perceive as fundamental to the internet (Cohen-Almagor, 2012). This perception is grounded in the theory of democracy,^3^ and particularly in contemporary conceptualizations of public discourse, which contend that the internet has become a “public sphere” (Aharony, 2012; Weber, 2013). Habermas (1989) classically defined the public sphere an intermediate space between the state and civil society, where all people have equal power and status. This egalitarian space creates an “ideal” discourse, where everyone can—yet must not feel pressure to—express his or her views, aspirations, and needs; ask questions; and raise criticism. Thus, according to the view of the internet as a public sphere, UGC platforms such as comment sections on online content sites should constitute a democratic arena of speech, where symmetry, reciprocity, and egalitarianism are maintained (e.g., Gervais, 2015). The value of upholding internet platforms as egalitarian spaces becomes even more salient when there is a risk of bias in determining which users get to express their views, as is the case for many content moderation practices (O’Brien, 2019; Riedl et al., 2020). In particular, content moderation might lead to underrepresentation of voices of specific individuals, arguably from marginalized and previously excluded groups, who might resort to commenting in an uncivil manner, for example, to express anger and rage over injustices (e.g., Clark, 2016; Traister, 2018). Considering that the ability to serve as a forum for criticism of all parts of the population is a key element in journalism (Kovach & Rosenstiel, 2007), content moderation, which compromises this ability, might also be dissonant with the philosophy of website owners and content creators.

However, simply providing an online space for unrestrained speech does not necessarily guarantee egalitarian freedom of expression. In general, as observed by Velasquez (2012), the extent to which individuals participate in online conversations is not only innately driven but is also influenced by many contextual factors, including characteristics of the content at the focus of discussion. Moreover, when engaging in online conversations, people tend to self-sort into groups corresponding to specific themes, political ideologies and attitudes, which prevents them from dealing with diversity and divergent views (Gervais, 2015). Particularly relevant to our context, several studies suggest that the interplay between free expression and incivility can create a paradox, such that when participants are free to behave uncivilly or antisocially, communication is ultimately damaged—as individuals who feel harmed by uncivil behavior no longer feel comfortable expressing themselves (Anderson, et al., 2014).

In this paper, we propose a means of resolving the tension between websites’ responsibility to mitigate uncivil behavior and the value of freedom of expression. Specifically, we explore an approach that does not eliminate uncivil content but instead reduces its capacity to cause harm. Our approach is grounded in the ideas that (i) incivility may be contagious, such that exposure to an uncivil comment can trigger subsequent uncivil comments; and (ii) the extent to which an uncivil comment is contagious depends on the positioning of the comment in a list of comments. We provide our theoretical rationale for these ideas in the following subsections.

### Contagion in Online Incivility

The term "contagion" is widely used in the domain of biological sciences to refer to the spread of disease through close contact among individuals. Yet, ideas and behaviors can also spread among individuals in a contagion-like fashion (Angst et al., 2010). This spread occurs because people consistently rely on information from their environment to interpret others’ behavior (e.g., Banerjee, 1992), create their beliefs, and make decisions by doing what others are doing regardless of their knowledge of the situation (Asch, 1951). In this paper, we use the term "[social] contagion" as an umbrella term for the various diffusions of ideas or practices through social systems (Angst et al., 2010).

A large body of literature stresses the automatic nature of social cognition, showing that the effects of social judgment and behavior occur without deliberate reflection (Adolphs, 2009; Frith & Frith, 2008; Marsden, 1998). Studies of social contagion have been conducted in multiple contexts, including the spread of information (Mønsted et al. 1998), emotions (Barsade, 2002; Cheshin et al., 2011; Kramer, 2014; Neumann & Strack, 2000), health behavior (Centola, 2011; Cohen-Cole & Fletcher, 2008) and unethical behavior (Den Nieuwenboer & Kaptein, 2008; Heet al., 2019; Liu et al., 2015; O’Fallon and Butterfield, 2011). In particular, when it comes to negative behaviors, evidence suggests that contagion may be simple, based on a single exposure, and without awareness. Foulk et al. (2016) developed a model of such behavioral contagion, an extension of Barsade’s (2002) model of emotional contagion. In a study on workplace rudeness, the authors claimed that negative behavior is ‟like a common cold;” that is, negative behaviors are common, easily caught, even after just a single exposure from nearly anyone.

In line with these findings, practitioners (Chen & Pain, 2017) and scholars (Su et al., 2018) have recently raised the possibility that online commentary may be a contagious behavior, such that exposure to incivility triggers uncivil behavior in others. Chen and Pain (2017) interviewed journalists who stated that, when possible, they try to be the first commenter, to inspire subsequent civil conversation. In the context of online deliberation, Suh et al. (2018) found that the initial comments inserted by the authors influence the quality of the subsequent discussion. In contrast, Rösner and Krämer (2016) failed to find similar results. Indeed, thus far, empirical studies have produced inconclusive findings regarding the extent to which online incivility is contagious (e.g., Gervais, 2015; Han et al., 2018; Kim et al., 2021; Rösner & Krämer, 2016; Ziegele et al., 2018).

Herein, we propose a means of reconciling these findings by suggesting that not all exposures to incivility are equally contagious. Instead, we identify a novel feature that might affect the extent to which a particular uncivil comment is contagious (i.e., likely to trigger additional uncivil comments)—namely, the comment’s serial position in a list of comments.

### Serial Position Effect

In proposing that an uncivil comment’s serial position may affect the behavior of users exposed to that comment, we build on the literature on the serial position effect (Bar-Hillel, 2015). This literature states that, when people pursue information in a sequential manner—e.g., by viewing a list of items—the serial position of an item in the list can affect various aspects of information processing. Specifically, research in this vein has demonstrated that an item’s serial position can affect the extent to which it is retained in memory (Bruce & Papay, 1970; Duncan & Murdock, 2000; Tse and Less, 2001), attitude formation regarding the item (Asch, 1946; Buda & Zhang, 2000; Haugtvedt & Wegener, 1944), and choice of items associated with the information (Bergus et al., 2002; Lohse, 1997).

The literature on serial positioning has identified two main types of effects. The first is the primacy effect, which occurs when the first few items of a list or a sequence are chosen or remembered over all other items (Anderson, 1965; Asch, 1946; Krosnick & Alwin, 1987; Purnawirawan et al., 2012). The other is the recency effect, which occurs when the last items of a list or a sequence are chosen or remembered over all other items (Krosnick et al., 1990; Purnawirawan et al., 2012; Smith & Vogt, 1995; Wang, 2011; Ying & Chung, 2007).

Primacy and recency effects have the potential to emerge in online environments, given that webpages are essentially structured as lists, in which users must scroll down to view additional information. Indeed, several studies lend empirical support to this idea (e.g., Nazlan et al., 2018). Many of these studies specifically affirm the primacy effect in information search, showing that the higher a link’s position in a list of links, the higher the probability that information searchers will click on the link (Agarwal et al., 2011; Ansari & Mela, 2003; Drèze & Zufryden, 2004; Ghose et al., 2013, 2014; Jeziorski & Moorthy, 2018; Rutz et al., 2012; Wang et al., 2019). For example, Ansariand (2003) found a negative correlation between link order and response rate, indicating that the effectiveness of links decreases as the link appears later in an email. These findings have had concrete effects on how website owners lay out their content; for example, it is common for managers and website designers to place their most desirable links toward the top of a web page or email and their least desirable links toward the bottom of the web page or email (Murphy et al., 2006).

Researchers have devoted somewhat less attention to recency effects in online settings, that is, how users respond to the last information encountered. Works that do consider recency effects point to a dual primacy-recency effect, in which the first and the last elements in a list result in a higher impact than all other items (Ert & Fleischer, 2016; Kolomiiets et al., 2016; Murphy et al., 2006; Purnawirawan et al., 2012). For instance, Purnawirawan et al. (2012) and Kolomiiets et al. (2016) examined the impact of negative reviews on perceived usefulness and buying intentions. In this context they found a “wrap effect”, implying that the impact of negative reviews that appear on both the top and bottom of a review thread is higher than that of negative reviews that appear on the top of a thread only. Studies in other domains lend further support to this dual primacy–recency effect (Baucells et al., 2011; Dayan & Bar-Hillel, 2011; Wedel & Pieters, 2000). For example, Baucells et al. (2011) found that investors update their reference price by combining the first and the last prices of a time series, with intermediate prices receiving smaller weights.

To our knowledge, researchers have yet to explicitly investigate how the serial positioning of information such as user-generated comments plays into online incivility. Still, some studies provide indirect evidence for primacy effects in the context of online incivility, showing that comments at the head of a list can influence the civility of subsequent comments (Chen & Pain, 2017; Suh et al., 2018)—albeit without comparing these effects to those of comments in the middle or at the end of the list.

Given these findings, combined with the broader evidence regarding serial position effects in online settings (Agarwal et al., 2011; Jeziorski & Moorthy, 2018; Kolomiiets et al., 2016; Purnawirawan et al., 2012), it seems plausible that primacy or recency effects might be observed in the extent to which uncivil comments trigger subsequent uncivil comments. The presence of such effects would further imply that uncivil comments positioned in the middle of a list are *less* likely to spur uncivil commenting compared with similar comments positioned at the beginning or the end of the list. Thus, comment repositioning may serve to curb online incivility without eliminating existing uncivil comments.

Considering the potential positive outcomes of a comment repositioning process, in this research we first ask the basic question: Does the serial positioning of an uncivil comment affect the extent to which subsequent comments tend to be uncivil? Notably, if such an effect is indeed at play, it could contribute to explaining the inconsistencies in prior findings regarding whether or not incivility is contagious (Rösner & Krämer, 2016; Suh et al., 2018). If serial positioning indeed affects civility, another question arises as to which comment position is most associated with further comments’ incivility. We explore these questions in three studies: one based on observational data and two controlled lab studies. On the basis of our findings, we seek to identify the optimal serial layout of a comment thread so as to minimize the contagion of online incivility.

In addressing our research questions, we acknowledge the possibility that, in attempting to resolve one ethical quandary through comment repositioning, we may be inadvertently introducing another: namely, a chilling effect, in which valuable types of speech are negatively influenced by actions aimed at restricting other types of speech (Kendrick, 2012; Schauer, 1978). In our case, this could mean that positioning uncivil comments in the middle of a comment list might cause commenters to limit or otherwise modify their contributions of civil comments. We note, however, that one study that empirically measured the chilling effect in the context of online reviews found that different types of restrictions on review writing elicited only a slight and desirable impact on the style and tone of the reviews, and that they did not have any effect on the content of those reviews (Bedi, 2021). We further explore the possibility of a chilling effect in one of our lab studies.

## Observational Study

This study relied on analysis of observational data from a national online content website, in which a subset of posted comments was not subject to moderation.

### Data Collection

We obtained consent from the administrators of an Israeli national content website to download articles and their corresponding comments for the purpose of this study. We collected via a designated crawler all articles and comments that had been posted on weekends between January 19, 2019 and June 1, 2019. Our focus on weekends was motivated by the fact that, according to our communication with the website’s administrators, comments posted to the website on these days were not subjected to moderation: Though the website had an active moderation process, in which uncivil comments were rejected by a single moderator, the moderator did not work on weekends, and the website’s policy was to enable comments to be posted during these times without undergoing any manual filtering.

The website’s reach on weekends was 5% of the population of the country. Of note, comments posted on the website were all presented at once, in a single list, meaning the user could view all comments by scrolling, and no comments were “hidden” behind a link. Comments were numbered according to the order in which they were posted and appeared in reverse chronological order, with the exception of comments that were posted as replies to existing comments; each reply appeared as part of a thread beneath the original comment. Such reply threads were very rare, however (mean thread length = 1.55; comments median length = 1 comment). Accordingly, in our analysis, we focused only on the first level of comments. To be included in our analysis, an article had to have a minimum of 30 comments (an indication of interest in the topic/article). At the end of the data collection period, our sample included 100 articles and their corresponding comments (*n* = 6510).

### Content Analysis

We used the following two-stage procedure to label each comment as civil or uncivil. In stage one, following the Intercoder Reliability (ICR) method (O’Connor & Joffe, 2020), three external expert reviewers, who were unaware of the goals of the study, were independently presented with 10% (*n* = 600) of the sample comments in random order. Each reviewer was asked to classify each assigned comment as either civil (1) or uncivil (0) using a civility scheme based on the literature (Coe et al., 2014; Sydnor, 2018; see coding scheme in Appendix A). Uncivil coding required the comment to include one or more of the following attributes (based on Papacharissi, 2004; Rossini, 2020; Stroud et al., 2015): (1) Name-calling, (2) Aspersion, (3) Lying accusations, (4) Vulgarity, or (5) Pejorative for speech. Reliability of the incivility judgments by the three independent coders was of a satisfactory level (Krippendorff α = 0.64^4^; Cronbach’s α = 0.838). In stage two, the remainder of the sample was labeled by one of the three coders (following the practice discussed in O’Connor & Joffe, 2020). Overall, the three coders labeled 18.8% of the comments in our dataset as uncivil. This percentage is aligned with the findings of Coe et al. (2014), discussed above.

### Analysis of Serial Position Effects

We then proceeded to evaluate the associations between the civility ratings of comments located in specific serial positions and the civility ratings of subsequent (“focal”) comments. In general, we assigned each comment an index according to its position in its corresponding list (e.g., comment 1 is the comment posted first in its list, comment 2 is the comment posted second, etc.). We then defined a comment’s serial position (“first”, “last” or “middle”) as follows. A comment was defined as being “first” if it was the first comment posted in its list. The identity of the “last” comment in the list, however, was dependent on the index of the focal commenter observing the list. For example, for the fourth commenter, running comment number 3 was last. For the fifth commenter, comment number 4 was last. Similarly, a “middle” comment was defined as any comment that, from the focal commenter’s perspective, was not first or last in its list. A contingency table revealed that a civil comment was followed by another civil comment with a probability of 0.83, and by an uncivil comment with the complementary probability of 0.17. An uncivil comment, on the other hand, was followed by a civil comment with a probability of 0.73, and by an uncivil comment with a probability of 0.27 (${x}^{2}\left(1\right)=39.82,p<.001$). The finding that an uncivil comment was more likely than a civil comment to be followed by an uncivil comment is compatible with previous works that have noted that incivility is contagious (e.g., Kim et al., 2021; Ziegele et al., 2018).

Next, we examined the primacy effect—the effect of the first comment in each article on the prevalence of uncivil comments in that article. To this end, we used a Random-effects model. Random-effects models are often employed when there are repeated observations on the same subject or when the data are clustered (e.g., Agresti et al., 2000; Jank & Yahav, 2010). Since we have many comments on the same article, we added a random effect on the articles’ identifiers. By doing so, we essentially allowed for different incivility intercepts for each article. Equation 1 describes the model we fitted, with *i* being a commenter indicator, and *j* being an article indicator. *P*_*i*_ in the equation refers to the civility rating of the first comment to which commenter *i* was exposed (civil vs. uncivil), such that the coefficient on *P*_*i*_ captures the primacy effect. The primacy effect was found to be significant (*p* = 0.04), with an uncivil first comment increasing the odds of incivility by 42% (β = 0.35, odds ratio = 1.42).

$$(1)\,logit({p\left(comment\, is\, uncivil\right)}_{ij})= {\beta }_{0 }+{\beta }_{1}\times {P}_{i}+ {article}_{0j}+{e}_{ij}$$

To rule out the alternative explanation that the effect observed was driven by unobservable characteristics of the articles, regardless of the civility rating of the first comment, we repeated the random-effects analysis, this time evaluating the effect of a *randomly* selected comment on the rate of incivility (see Eq. 2, with *R*_*i*_ being the civility rating of a randomly selected comment). The effect was found to be insignificant (*p* = 0.14) and smaller (β = 0.25, odds ratio = 1.28), thus refuting the alternative explanation. These findings suggest that a user’s likelihood of posting an uncivil (as opposed to civil) comment is greater when the *first* comment in the list is uncivil rather than civil. The results are summarized in Table 1.

**Table 1:** The effect of the civility rating of the first comment versus that of a random comment on commenters’ propensity for incivility in the main experiment

	Logistic Model									
	Primacy effect					Alternative model				
Effect	Estimate	*SE*	95% CI		*P*	Estimate	*SE*	95% CI		*p*
			*LL*	*UL*				*LL*	*UL*	
Fixed effects										
Intercept	1.289	0.155	0.985	1.593	< 0.001	1.380	0.145	1.096	1.664	< 0.001
P_*i*_ (primacy: first comment’s tone)	0.356	0.175	0.013	0.699	0.043					
R_*i*_ (random comment's tone)						0.255	0.169	− 0.076	0.586	0.14
correlation of fixed-effects	*− 0.876*					*− 0.853*				
Random effects										
Between-study variance	0.315	0.56	-0.783	1.413	> 0.05	0.329	0.573	-0.794	1.452	> 0.05

$$(2)\, logit({p\left(comment\, is\, uncivil\right)}_{ij})= {\beta }_{0 }+{\beta }_{1}\times {R}_{i}+ {article}_{0j}+{e}_{ij}$$

To examine the recency effect—the extent to which a person is likely to post an uncivil (vs. civil) comment when the *last* comment he or she observes is uncivil (vs. civil)—we conducted an autocorrelation test. This test examined the correlation between the civility rating (civil vs. uncivil) of each focal comment (*m*) and the civility rating of the last comment published before that comment (*m-*1). We also examined the correlation between comment *m*'s civility rating and the civility rating of each comment preceding *m* with lag *l*: *ρ(m, m-l)*. Together, these comparisons reveal how a focal commenter’s likelihood of posting uncivil content is affected by incivility posted by each of the last *l* commenters. The analysis revealed a significant decreasing autocorrelation of incivility: 10% (*p* < 0.001) in lag 1 (correlation between the focal comment and the last published comment), 5% (*p* < 0.001) in lag 2, 4% (*p* < 0.001) in lag 3, and no autocorrelation thereafter. This result implies that a user’s likelihood of posting an uncivil (as opposed to civil) comment is greater when the *last* (up to three) comment(s) in the list are uncivil rather than civil, yet is not significantly affected by older comments (preceding the focal comment by more than three). This result, together with the primacy effect discussed above, implies that the comments positioned at the edges of a list have the highest impact on the following commenter’s tone. Thus, a website that seeks to prevent commenters from posting uncivil content may benefit from “hiding” uncivil comments in the middle of a comment list, while “padding” the thread with civil comments at the top and bottom.

It is worth noting that although the comments were numbered based on their time stamps, they were actually sorted in reverse chronological order. Therefore, some users may have perceived the first comment posted as the last one in the thread, and vice versa. In such a scenario, the analyses presented in this section would be reversed, such that the effect of primacy would become the effect of recency, and vice versa. However, it is important to emphasize that both effects are significant and in the same direction, regardless of the interpretation. Our controlled studies further address this possible confound, as elaborated next.

## Overview of Randomized Controlled Studies

To shed further light on the phenomena revealed in our observational study, we conducted two randomized controlled studies. Before initiating these studies, we carried out a pre-test to examine factors that are unrelated to the order of a comment thread that might contribute to online incivility, such as age, gender (Alonzo and Aiken, 2004; Hmielowski et al., 2014; Vochocová & Rosenfeldová, 2019), the topic of discussion (Stroud et al., 2015; Su et al., 2018; Ziegele & Jost, 2020), and the opinion about the subject of discussion (Gervais, 2015). This test established that gender, and the topic discussed affect the probability of writing in an uncivil manner, implying that our subsequent experiments should be balanced based on these factors (see Appendix B for the full report on the pre-test).

Next, we carried out a pilot study to obtain a general picture of how the serial positioning of civil versus uncivil comments affects various aspects of users’ engagement with those comments and with the corresponding content. Such effects should be taken into account when considering introducing a comment reordering scheme, and they may have direct methodological relevance to the analysis of incivility contagion.

Finally, we carried out our main experiment, focusing on the effects of a platform’s comment layout on incivility contagion. Our pilot study and main study are described in detail below.

## Pilot Study

The pilot study aimed to achieve the following specific goals. First, it sought to measure how the serial positioning of civil versus uncivil comments affects users’ general tendency to post comments themselves. From a methodological perspective, such an effect could have implications for subsequent analysis of users’ tendency to express themselves civilly or uncivilly in the comments they post. An investigation of users’ general commenting tendencies can further provide important practical insights. For example, it can indicate whether comment reordering risks introducing a chilling effect, as discussed above (Bedi, 2021; Kendrick, 2012; Schauer, 1978). Moreover, commenting likelihood serves as an indicator of user engagement, which website owners consider to be a positive outcome and seek to promote (Borah, 2014; Brooks & Geer, 2007). Second, we investigated how the serial positioning of uncivil comments affects readers’ attention, towards evaluating, for example, whether uncivil comments that are placed in the middle of the comment thread are simply unseen and unread. Such a phenomenon could explain any attenuating effects of comments’ serial positions on subsequent incivility—yet, it might raise concerns regarding whether ‘hiding’ comments is akin to deleting them. To evaluate users’ attention, we measured participants’ recognition of comments they had seen, in accordance with findings that heightened focus on information manifests in an enhanced capacity to recognize the information (LeBoeuf et al., 2014). Third, to further delve into user engagement in response to exposure to civil versus uncivil comments, this study analyzed the extent to which participants like or dislike each type of comment.

### Method

#### Participants

Our experiment involved 1,004 participants (μ_*age*_ = 37.54, 47.4% female) from the United States, recruited from Prolific in exchange for monetary compensation (for full demographics, see Appendix C, Table C.1). Prolific is an online labor and research platform, considered to be an equal alternative to the more popular Amazon Mechanical Turk, with the advantage being that Prolific workers are naiver when it comes to experimental tasks, resulting in more authentic answers (Peer et al., 2017). Each participant was randomly assigned to one of four conditions corresponding to different modes of exposure to civil versus uncivil comments, in a between-subjects design.

#### Procedure

Each participant was presented with an article that referred to a controversial topic (adding Trump’s face to Mount Rushmore). We selected this article from a set of several real articles that we had previously identified as controversial, based on the fact that they had generated hundreds or even thousands of responses (both civil and uncivil) on social networks (see “Main Experiment” section for additional details). The article was designed to resemble an article on a Facebook page, followed by six comments, similar to what one might encounter in a typical Facebook post (see Appendix C).^5^ The number six was chosen based on Murphy et al. (2006) to ensure that, for common screen and browser configurations, participants would see all comments on one page. The use of more comments could have made it necessary for some users to scroll down to view all comments, potentially confounding our controlled setup. The comments used in our experiment were taken from a set of actual comments that social media users had posted in response to the focal article.

We chose four civil comments and two uncivil comments, which participants viewed in the following configurations, corresponding to their experimental conditions: (1) ‘civil’ condition (control-filtering): four civil comments; this configuration represents a filtering process in which all uncivil content is removed following the common practice (we note that, in practice, less common or more subtle forms of incivility might be more difficult to detect and therefore usually remain in the thread; Coe et al., 2014); (2) ‘uncivil primacy’ condition: two uncivil comments at the top of the thread followed by four civil comments; (3) ‘uncivil recency’ condition: four civil comments followed by two uncivil comments; and (4) ‘uncivil middle’ condition: two civil comments followed by two uncivil comments and two civil comments. Participants were instructed to read the article and the comments and post their own comments on the article (this was done to disguise the real purpose of the study).

Next, we measured the effect of experimental condition (comment configuration) on participants’ general tendency to post comments. To this end, we asked participants to report whether they would comment if they were to read the post on social media (Yes/No).

We subsequently assessed the effect of experimental condition on readers’ attention and comment recognition. Specifically, we randomly presented participants with four uncivil comments (in random order): two of the uncivil comments were taken from the comments that were posted in response to the article, and two additional uncivil comments did not appear in the article’s comment section.^6^ For each comment, we asked participants to indicate if they had read it before (Yes/No) (adapted from Hawkins & Hoch, 1992).

Then, we presented each participant with the original six comments (two uncivil and four civil) one by one in random order and asked them to indicate whether they liked or disliked each comment (Yes/No). Finally, participants completed demographic measures referring to their age, gender, education level, marital status, and employment status.

### Results

#### Verification of Homogeneity across Conditions

We identified no differences across conditions in terms of demographics (gender: χ^2^(6) = 2.75, *p* = 0.839; age: *F*(3,999) = 0.558, *p* = 0.643; education level: χ^2^(18) = 16.65, *p* = 0.547; marital status: χ^2^(12) = 13.71, *p* = 0.320, and employment status: χ^2^(18) = 13.64, *p* = 0.752), thus verifying our random assignment.

#### Analysis of Serial Position Effects

We conducted a Chi-square test to analyze the total impact of the serial position of uncivil comments on participants’ intentions to post comments on social media; the results indicated that this impact was not significant (${\chi }^{2}\left(3\right)=2.66, p=.447)$. We subsequently carried out Z-proportion tests with the ‘uncivil middle’ condition serving as a reference (8.3% of participants in this condition reported that they were likely to post a comment). This analysis indicated that, across the three remaining conditions, participants’ reported likelihood of posting a comment was similar to that obtained in the ‘uncivil middle’ condition (‘civil’ condition: 10.4% likely to post, *Z* = − 0.79, *p* = 0.43; ‘uncivil recency’ condition: 10.1%, *Z* = − 0.69, *p* = 0.49; ‘uncivil primacy’ condition: 12.7%, *Z* = − 1.61, *p* = 0.11). These results suggest that the serial positioning of civil versus uncivil comments does not affect users’ general tendency to post comments themselves.^7^

To examine whether participants’ experimental conditions affected comment recognition, we calculated the proportion of correctly recognized original uncivil comments (0 = not recognized, 1 = recognized) for each condition in which participants had been exposed to uncivil comments. A Chi-square test indicated that the effect of experimental condition on this measure was insignificant (‘uncivil primacy’ condition: 78.2%, ‘uncivil recency’ condition: 75.2%, ‘uncivil middle’ condition: 75.5%; ${\chi }^{2}\left(2\right)=1.49$, *p* = 0.474). This result suggests that any serial positioning effects are unlikely to be driven merely by differences in the level of attention paid to uncivil comments in certain positions.

To examine whether the civility of a comment affected its likelihood of being liked, we calculated the averaged “likes” rate for uncivil and civil comments. A Z-proportion test indicated that uncivil comments received significantly more “likes” (46.4%) than civil comments did (43.7%, *Z* = − 1.98, *p* = 0.024).

## Main Experiment

Our main experiment aimed to study, in a controlled environment, how the serial positioning of civil versus uncivil comments affects the extent to which incivility is contagious. In this experiment, we exposed users to different configurations of comments and then asked them to post a comment themselves. We subsequently evaluated the civility levels of participants’ comments.

We note that the mandatory nature of comments (a choice we made to ensure sufficient data for analysis) did not enable us to condition our measurement of comments’ civility on participants’ decisions to comment in the first place. However, given the finding of the pilot study that comments’ serial positions do not influence users’ general likelihood of commenting, we assume that the conditional probability of commenting uncivilly, given that the user has chosen to comment, is equal to the unconditional probability of commenting uncivilly when comments are mandatory.

### Method

#### Participants

Our main experiment involved 1,000 participants (μ_*age*_ = 32.8, 50.2% female)^8^ from the United States, recruited from Prolific in exchange for monetary compensation. Each participant was randomly assigned to one of four conditions, in a between-subjects design, as elaborated in what follows.

#### Procedure

Each participant was presented with an article, which was randomly selected from a set of five possible articles (one of which was included in the pilot study; see Appendix C). Like the article used in the pilot study, each of these articles referred to a topic that we had previously identified as controversial (according to real responses to the article on social media).

As in our pilot study, for each article, we chose four civil comments and two uncivil comments that had originally been posted on social media in response to the article (see Appendix D). Likewise, the configuration of comments that each participant viewed corresponded to his or her condition, as follows: (1) 'civil' condition (control-filtering): four civil comments; (2) 'uncivil primacy' condition: two uncivil comments at the top of the thread followed by four civil comments; (3) 'uncivil recency' condition: four civil comments followed by two uncivil comments; and (4) 'uncivil middle' condition: two civil comments followed by two uncivil comments and two civil comments. Participants were instructed to read the article and the comments as they typically would and post their own comments on the article. We told the participants that former participants had written the comments, and that their comments might be visible to future participants. Lastly, participants stated their age and gender.

**Fig. 1:**
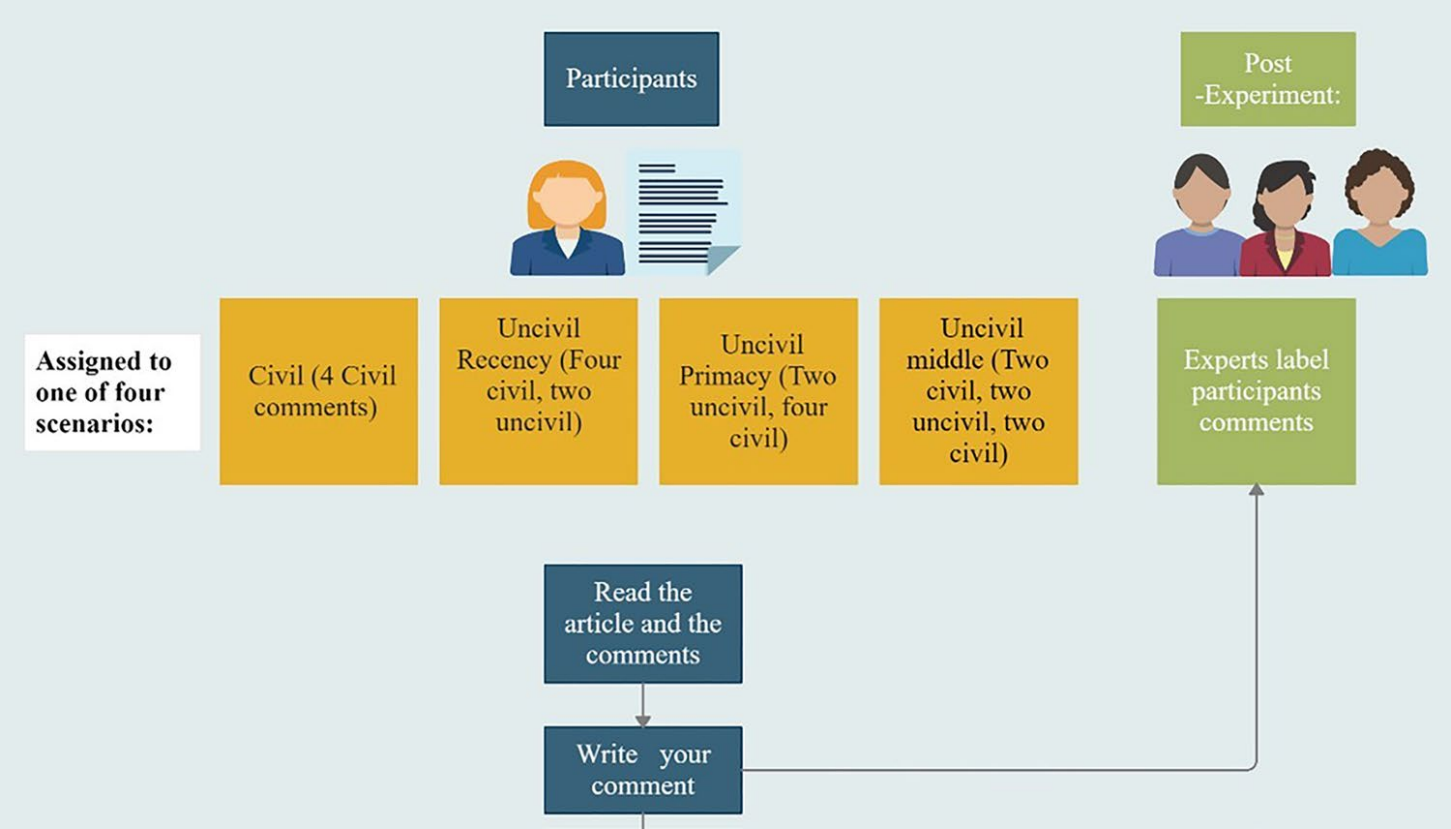
Schematic illustration of experimental procedure in the main experiment

After data collection was complete, participants’ comments were labeled by independent content coders as either civil or uncivil, according to the content analysis procedure described below. Figure 1 provides a schematic illustration of our experimental procedure.

#### Content Analysis

In accordance with the Intercoder Reliability (ICR) method (O’Connor & Joffe, 2020), three external expert reviewers, who were blind to the goals of the study, were independently presented with each of the 1,000 comments written by the participants in the experiment. Each reviewer was asked to classify each comment as either civil (1) or uncivil (0) using the same civility scheme as in the pilot study (Coe et al., 2014; Sydnor, 2018; see Appendix A).

The reliability of the three independent coders was not of a satisfactory level (Krippendorff α = 0.29). Thus, after the individual scoring, we carried out an intercoder reliability process in which differences in the civility/incivility classifications between reviewers were discussed. First, each expert presented their justification and interpretation of the civility scheme. Next, coders revised the coding of comments and converged to a single agreed-upon classification. There were five comments for which the coders could not reach an agreement regarding the classification. Therefore, these comments were excluded from the analysis (O’Connor & Joffe, 2020). Among the remaining 995 comments, 77.39% were classified as civil and 22.61% were classified as uncivil.

### Results

#### Verification of Homogeneity across Conditions

We identified no differences across conditions in terms of demographics (gender: χ^2^(8) = 6.77, *p* = 0.526; age: *F*(3,533) = 0.038, *p* = 0.99), thus verifying our random assignment.

#### Analysis of Serial Position Effects

We conducted a Chi-square test to analyze the total impact of experimental condition on the civility ratings of participants’ comments; the results indicated that this impact was significant (${\chi }^{2}\left(3\right)=8.68, p=.03)$. Next, we conducted Z-proportion tests, with the 'civil' condition serving as a reference. We found that compared with participants in the 'civil' condition (17.46% uncivil comments), participants in the 'uncivil primacy' condition were more likely to post uncivil comments (24.67% uncivil comments, *Z* = 1.98, *p* = 0.02), as were participants in the 'uncivil recency' condition (27.71% uncivil comments, *Z* = 2.74, *p* = 0.003). Participants in the 'uncivil middle' condition were not significantly more likely than participants in the 'civil' condition to post uncivil comments (20.65% uncivil comments, *Z* = 0.90, *p* = 0.18), suggesting that when uncivil comments are placed in the middle of the comment thread (as opposed to the beginning or the end), incivility is less likely to be contagious.

Towards further elucidating these apparent recency and primacy effects, we compared participants in the 'uncivil middle' condition with those in the 'uncivil recency' condition and found that the latter were more likely to post uncivil comments (*Z* = 1.84, *p* = 0.033). However, there was no significant difference between the 'uncivil middle' and the 'uncivil primacy' conditions in terms of the likelihood of posting uncivil comments (*Z* = 1.07, *p* = 0.14).

To further analyze how participants’ likelihood of commenting uncivilly was influenced by the positioning of uncivil comments in the list, we conducted a logistic regression. Specifically, as in our observational study, we used a random-effects model on the probability of commenting uncivilly as a function of the position, with a random effect on the article displayed. Equation 3 describes the model we fitted, with *i* being a commenter indicator, and *j* being an article indicator. *Position*_*i*_ in the equation refers to the position of the comments (with the ‘civil’ case as the reference category). The results, presented in Table 2, reinforce the recency and primacy effects (recency: β = -0.497, *p* = 0.038; primacy: β = -0.666, *p* = 0.005), and the non-effect of placing uncivil comments in the middle of the comments thread (*p* = 0.379).

**Table 2:** The effect of experimental condition on commenters’ propensity for incivility in the main experiment, with a random effect on the article

	Logistic Model				
Effect	Estimate	*SE*	95% CI		*p*
			*LL*	*UL*	
Fixed effects					
Intercept	1.881	0.508	0.885	0.877	0.0002
Condition: primacy	-0.497	0.239	-0.965	-0.029	0.038
Condition: recency	-0.666	0.236	-1.129	-0.203	0.005
Condition: uncivil middle	-0.215	0.245	-0.695	0.265	0.379
Random effects					
Between-study variance (article)	1.116	1.1056	-1.051	3.283	> 0.05

$$(3)\, logit\left( {p\left( {comment\,is\,uncivil} \right)_{{ij}} } \right) = \beta _{0} + \beta _{1} \times Position_{i} + article_{{0j}} + e_{{ij}}$$

## Discussion

This research sought to address the ethical tension that arises between a website owner’s responsibility to ensure a civil environment for users and the wish to uphold the value of freedom of expression online. To this end, we explored a novel alternative to traditional comment moderation practices—an approach in which uncivil comments are not eliminated but instead are repositioned strategically within the list of comments, thereby reducing their capacity to cause harm by triggering further incivility. Our results lend support to the idea that online incivility can be—but is not always—contagious (e.g., Gervais, 2015; Kim et al., 2021; Rösner & Krämer, 2016; Ziegele et al., 2018), and that the extent of contagion is subject to serial position effects. Table 3 summarizes the studies.

**Table 3:** Summary of the studies

Study	Method	Independent Variable	Dependent Variables	Main Findings
Observational study	Analysis of data from an online content website	Comment’s serial position	Incivility likelihood	The likelihood of writing an uncivil comment is higher when the first or the last comments in a list are uncivil
Pre-test	Experimental between-subject design	Age, gender, the topic discussed, the opinion about the subject of discussion	Incivility likelihood	Gender and topic affect incivility likelihood (and should be controlled in experiments)
Pilot study	Experimental between-subject design	Comment’s serial position	User engagement: commenting likelihood, attention, comment liking	Comment position affects neither commenting likelihood nor attention to uncivil comments (which therefore do not confound the main experiment’s results) Uncivil comments receive more “likes” than civil comments
Main experiment	Experimental between-subject design	Comment’s serial position	Incivility likelihood	The likelihood of writing an uncivil comment is higher when the first or the last comments in a list are uncivil

More specifically, our observational study revealed that exposure to an uncivil comment that appears either first or last in the thread (as opposed to a civil comment that appears in these positions) makes users more likely to post an uncivil (rather than civil) comment themselves. Our main lab experiment enabled us to further establish these primacy and recency effects, while controlling the user’s overall experience and manipulating the order of comments to which he or she was exposed. The validity of this experiment was supported by a pilot study, which enabled us to verify that adjustment of the serial positioning of uncivil comments can indeed be considered as an ethical and practical alternative to manual comment moderation. First, that study showed that the serial position of uncivil comments does not affect users’ general likelihood of posting a comment. Accordingly, it seems that the use of a rearrangement process has a minimal risk of creating a chilling effect in which users avoid participating (Kendrick, 2012; Schauer, 1978). However, it is important to note that the chilling effect is not only about actual silencing of voices, but also about the perception of the policy. Even if the policy does not result in silencing voices, if people believe it does, then there could be a chilling effect. Therefore, it is crucial for companies to do a good job explaining the policy, how it works, and how it does not silence voices.

Moreover, our pilot study ruled out the possibility that the serial position effects we observed resulted from a lack of attention paid to uncivil comments in the middle of the thread—suggesting that our approach does not prevent commenters’ voices from being heard.

Our main experiment further enabled us to discern nuances of the primacy and recency effects. Specifically, we found that exposure to uncivil comments located at the bottom of the list (as compared with the top or the middle) had the strongest influence on further incivility. The latter result diverges somewhat from findings of studies in other online contexts (e.g., perceived usefulness of online reviews), which suggest that primacy and recency effects are comparable in magnitude (Dayan & Bar-Hillel, 2011; Ert & Fleischer, 2016; Kolomiiets et al., 2016; Murphy et al., 2006; Purnawirawan et al., 2012; Wedel & Pieters, 2000). This difference might stem from the fact that in comment sections, it is common for users to write their own comments directly after the last comment (i.e., most recent). Thus, the most recent comment may elicit the most top-of-mind awareness during the act of writing one’s comment.

### Practical and Ethical Implications

Together, our findings enable us to propose a practical solution for curbing online incivility in the comment section of a content website: ensuring that civil comments occupy the top and bottom positions of the comment list, while positioning uncivil comments in the middle. Given prior findings (Coe et al., 2014), supported by our own observational data, that ~ 20% of comments online are uncivil, such rearrangement should indeed be possible. Moreover, this rearrangement could be carried out automatically, as identifying civil posts (and positioning them strategically) might be an easier task for automatic classification as compared with parsing the 'grey area' of incivility and determining which comments should be deleted. Accordingly, this rearrangement process would require limited intervention of the website’s moderator, and thus be cost-effective, while also allowing for freedom of speech—potentially enabling ambiguous comments to be retained, without risking further incivility. Notably, Gonçalves et al. (2021) have shown that participants in online platforms are more accepting of algorithmic moderation than of human moderation. Thus, if an algorithm is used to rearrange comments in the manner we propose, users may ultimately perceive the procedure as relatively fair.

With regard to the design of an algorithm to identify and reposition uncivil comments, we emphasize that websites are likely to be able to achieve desired results using definitions of incivility other than the one adopted herein (namely, that of Coe et al., 2014). We selected our definition of incivility because of its popularity in other works in this domain. In practice, websites should fine-tune their definitions as to which comments should be considered uncivil (and thus moved to the middle of the list). Some platforms may wish for more freedom of speech at the risk of hurting people’s feelings, whereas others might take the opposite approach. We believe there is no ‘one size fits all’ approach when it comes to online incivility, and thus we refrain from making general claims about which types of comments should be considered uncivil.

We further acknowledge that, though we believe our approach is more ethical than classic moderation in terms of ‘giving voice’ to internet users, there are other ethical alternatives to comment moderation. Interactive approaches (e.g., Stroud et al., 2015), which aim to actively engage users and to encourage them to ‘improve’ their comments instead of repositioning or deleting them, might be considered particularly democratic. However, they cannot solve all moderation issues. First, it may be technically infeasible and costly to employ human workers to discuss, solicit revisions for, and recheck 20% of the comments. Moreover, effective interactions with commenters may require website representatives, as well as commenters, to identify themselves, potentially raising privacy risks—which constitute a separate ethical concern (particularly for commenters who prefer to remain anonymous). Finally, interactive approaches may simply not always work: Some commenters might wish to be uncivil and respond negatively to any attempts at mediation or interaction.

### Challenges and Limitations

It is important to acknowledge certain challenges with regard to the execution of the comment rearrangement process we propose. In particular, the feasibility of this approach is dependent on the presence of multiple comments, at least some of which are civil. If only one comment has been posted, for example, and that comment is uncivil, it is impossible to use a repositioning approach to prevent that comment from triggering further incivility.

Moreover, even when a rearrangement process is feasible, our findings regarding the recency effect raise certain complications. Specifically, whereas it is straightforward to determine which comment a user will view first, it is challenging to know which comment he or she will view last—as a user might not scroll through an entire comment thread. One option is to adopt a design that shows only a few comments at any given time (with the option, e.g., to "show X more comments"). This setup provides the website designer with control over the comments to which the user is exposed, as well as their order. As noted in the introduction, some social media platforms (e.g., Facebook) use a similar approach to comment presentation, albeit not necessarily for the purpose of mitigating incivility. Another option might be to make sure that a 'civil' comment is visible adjacent to wherever the user writes their own contribution, to make certain that the last comment a user observes does not contain any incivility.

It is also important to consider whether—as in the case of any moderation procedure—the comment rearrangement we propose might negatively affect the quality of the website and its level of interest to users. Past research has found mixed results regarding the impact of incivility on users’ engagement. Specifically, several studies reported a positive association between willingness to engage and exposure to uncivil commentary (e.g., Borah, 2014), whereas others reported opposite findings (e.g., Muddiman et al., 2020). The results of our pilot study suggest that the order of comments to which users were exposed did not reduce participants’ levels of engagement with the content, as reflected in their intention to post their own comments, as well as in their attention to the comments they read. However, we also found that uncivil comments were better liked than civil comments. This finding may pose a challenge to platform managers who wish to promote liked posts, on the one hand, but maintain a civil atmosphere in their platform, on the other hand. Specifically, the desire to increase user interest may spur managers to highlight and therefore promote uncivil-liked comments to the top of the comment list. However, the ethical responsibility to create a safe and civil environment for users may motivate the opposite action, namely position of uncivil comments in the middle of the thread.

Our research is not without limitations. We focused our study on comment sections, which are popular platforms for UGC, and which websites can typically moderate and control. However, once a website’s content is shared elsewhere—e.g., a New York Times article that is posted to a social media platform, such as Facebook—the content-generating website may no longer be able to control the discussion. Moreover, it is unclear to what extent such websites, which depend on social media to reach large audiences, have leverage to convince powerful social media platforms to change their moderation policies or community guidelines to improve civility. Thus, if a media outlet wishes to achieve control over the level of civility in discussions related to its content, its best option may be to encourage its user community to engage in discussions on its own turf—where it can also practice such re-ordering.

Beyond comment sections on content websites, our approach may also be applicable to social media websites (though, as noted above, such websites implement their own complex procedures for determining comment order). In social media feeds, featured content is individualized, and news stories are curated by the platform’s algorithms. Such algorithms may rely on multiple criteria to choose what content to promote—and incivility can be one such criterion.

Moreover, inside a given discussion thread in response to a particular social media post/tweet, the discussion itself is usually not provided in full; rather, it is common that certain "featured" comments receive the most exposure, whereas other comments are more difficult to access. Though the focus of this practice is not necessarily to mitigate exposure to incivility (but instead, e.g., to increase engagement), it provides a useful mechanism for platforms that might wish to adopt our proposal to hide in the middle (or in this case, to provide less exposure to) comments identified as uncivil.

While our research was based on standard practical methods, it is important to acknowledge their limitations on our methodological choices. In particular, we included configurations in our studies that reflected different sequences of uncivil comments, as well as a configuration that reflected a prevalent filtering process involving the deletion of such comments. Although this filtering process is a primary method used by many online platforms (e.g., Chen et al., 2011; O’Brien, 2019; Riedl et al., 2020), it resulted in fewer comments overall, which could have confounded some of our findings. Specifically, in addition to the civility of comments, their quantity may have also affected participants’ responses following the configuration with no uncivil comments, including their intention to write a comment and the extent of civility in their comments.

Furthermore, comments that appeared in a list were assumed to be independent of one another. This is true for some forms of UGC, for instance, online reviews or testimonials that do not inherently contain a conversational element. On some websites, however, comments may be part of a conversation and, as such, relate to other past comments, in a manner that requires some chronological ordering and indication of clear hierarchies—e.g., through the use of sub-sections or sub-threads. We note that in this case, our findings might be applied to determining the order of first-level threads, as well as of the comments in each sub-thread. Indeed, the contagious nature of incivility may suggest that replies to uncivil comments are likely to be uncivil themselves—highlighting the need to control first the serial positioning of the main threads, such that threads that begin with uncivil comments are located between other threads that begin with civil comments.

Relatedly, in this paper, we demonstrated that positioning uncivil comments in the middle of a comment list makes subsequent users less likely to post an uncivil comment themselves. However, we did not examine additional outcomes of such an intervention, including effects on attitudes and feelings of platform users. For example, uncivil comments on a particular issue may lead to perceptions of a greater risk associated with that issue, especially among those who a priori hold attitudes against the issue (Anderson et al., 2014). Thus, future research could aim to further explore the implications of the comment repositioning process on risk perceptions. In the current research, we considered the impact of the serial position of an uncivil comment on comment recognition, finding no such effect. Since negative (e.g., riskier) items are deemed more attention-grabbing than positive items (Fiske, 1980; Zajonc, 1968) and therefore more memorable (Dreben et al., 1979; Robinson-Riegler & Winton, 1996), a null memorability effect may suggest that risk perceptions are less affected by the serial position of uncivil comments. However, a more systematic investigation is needed to gain a better understanding of the influence of an uncivil comment’s position on users’ attitudes, judgments, and feelings.

## Appendices

### Appendix A: Coding Scheme

Incivility is defined as features of discussion that convey an unnecessarily disrespectful tone toward the discussion forum, its participants, or its topics.

Forms of Incivility:

**Table:**

Name-calling	Mean-spirited or disparaging words directed at a person or group of people (e.g., “the morons”)
Aspersion	Mean-spirited or disparaging words directed at an idea, plan, policy, or behavior (e.g., “Texting while driving is stupid”)
Lying accusations	Stating or implying that an idea, plan, or policy was disingenuous (e.g., “Americans have been screaming at the top of their lungs that this government is wrong, is corrupt, is lying, is deceiving the people, and is violating our constitution….”)
Vulgarity	Using profanity or language that would not be considered proper (e.g., pissed, screw) in professional discourse (e.g., “I hope the voters will … kick him out on his pompous ass next election”)
Pejorative for speech	Disparaging remark about the way in which a person communicates (mocking how others express themselves, e.g., “I am sick and tired of [them] throwing their tantrums....”)

### Appendix B: Pre-test of Incivility Contagion

In our pre-test, each participant was exposed to an article designed to resemble an article on a Facebook page, followed by four comments. The articles presented in the experiment (seven articles in total) were chosen with the aim of representing a variety of controversial topics, which generated hundreds or even thousands of responses (both civil and uncivil) on social networks. Comments for the experiments were gathered from the original comment sections of news articles, as posted on Facebook.

As part of an initial pre-processing step, we divided the comments into salient pro-topic and salient anti-topic comments. Comments in which the perspective was ambiguous were dropped from the sample. Next, we turned to the Amazon Mechanical Turk (MTurk) crowdsourcing platform, to label the civility of each of the remaining comments. Each comment was presented to a minimum of five workers, who rated it on a scale of 0 or 1 (with 0 = uncivil and 1 = civil). For each article, we selected the top four comments (rated according to the level of workers' agreement) corresponding to each of the following categories: uncivil pro-topic, uncivil anti-topic, civil pro-topic, civil anti-topic.

We recruited 1,484 adult US residents through MTurk. We excluded 69 participants who did not complete the experiment. Of the resulting sample (*n* = 1,415), 54% were male, 45% were female, and 1% preferred not to divulge their gender. Participants’ ages ranged from 18 to 78 (average = 36.4, SD = 12.08).

Participants first indicated their opinion on each of the seven topics (in randomized order for each participant) on a scale of 0 (strongly disagree) to 1 (strongly agree). Next, participants were each randomly assigned to read one of the seven articles and engage with a single setup of four comments on the article (civil/uncivil × pro-/anti-topic), whereby all four comments were the same in terms of civility level (civil vs. uncivil) and topic view (pro vs. anti).

Participants were instructed to read the article as well as the comments as they typically would, and then post their own comment on the article. After the participants finished writing their comments, they were asked to rate as either civil or uncivil each of the comments appearing in the discussion thread, as well as their own comments. A short demographic questionnaire accompanied the experiment.

To determine the civility level (i.e., civil or uncivil) of the participants' comments, as perceived by external readers (and, potentially, subsequent commenters), we turned to three independent coders, who were unaware of the purpose of the experiment. The coders were all US residents who were at least 18 years old. We informed them that they would see written comments posted by readers of articles appearing on a content website. All the coders labeled the comments as civil or uncivil (Cronbach's α = 0.78). The order of the comments was randomized for each coder to minimize the possibility of an order effect. The median of the three coders was used to classify the comments.

Overall, 24% of the comments were coded as uncivil. We examined the factors of incivility using the logistic regression in Eq. 4. We found a significant difference in the rate of incivility with respect to age, gender and the topic discussed. Further, uncivil comments increased the odds of incivility by 80% (β = 0.640, odds ratio = 1.90, *p* = 0.001) beyond age, gender, topic, article, and opinion on the topic of the article (for full statistics, see Table B.1). We further calculated an agreement measure. If the participants opinion on the topic was aligned with the commenters pro (anti) topic, then the participant agrees (holds the same opinion) with the commenters' opinion. The agreement with previous commenters was not found to significantly moderate the rate of incivility (*p* = 0.609).

$$(4)\,logit\left({p\left(comment\, is\, uncivil\right)}_{ij}\right)= {\beta }_{0 }+{\beta }_{1}\times Gender+{\beta }_{2}\times age+{\beta }_{2}\times aricle topic+{\beta }_{2}\times opinion on topic+{\beta }_{2}\times agreement+{\beta }_{2}\times comment civility+{e}_{ij}$$

Although previous studies found these factors to influence the rate of online incivility, controlling them (if possible) would not alleviate the contagious nature of incivility. Thus, our main experiment looks into the design of online platforms as a means to reduce incivility.

**Table B.1:** The effects of gender, age, article topic, opinion on the article topic, agreement, and comment civility on online incivility

Effect	Logistic model			
	Estimate	*SE*	*p*	*Odds ratio*
Gender	0.527	0.135	< 0.001	1.965
Age	-0.008	0.005	0.138	0.992
Article topic *			< 0.001	
Opinion on the article topic	0.217	0.198	0.273	1.242
Agreement	-0.008	0.267	0.975	0.992
Comment civility	0.636	0.134	< 0.001	1.889

*Each article had a different incivility rate

### Appendix C: Pilot Study

Below is the full description of the pilot study, as presented to the participants.

#### Instructions

Please read the post as well as the comments as you would normally do when reading a news article posted on Facebook. Please note that the comments were written by former participants, and your comments might be visible to future participants as well.

[article and comments].

**Table:**

Trump's face on Mount Rushmore: A White House aide asked (R) last year how an interested party might go about adding another giant presidential face to Mount Rushmore, a source told The New York Times South Dakota Gov. Kristi Noem was the first to publicly reveal Trump's desire to add his image alongside the 60-foot faces of Presidents George Washington, Thomas Jefferson, Abraham Lincoln, and Theodore Roosevelt carved into the mountain "Do you know it's my dream to have my face on Mount Rushmore?" he asked Noam, who was still in Congress at the time and running for governor. "I started laughing. He wasn't laughing, so he was totally serious," she recalled	
Civil: (1) We need to get rid of all the faces on that mountain and give it back to the Lakotas and honor the treaty (2) Such an event might not warrant a revolution, but I think we would see something like one (3) I can't wait to see his face up there. He's a better president than most of them (4) He has said he never jokes. This was not a joke	Uncivil: (1) JFC- being an ass isnt monument worthy. How about a nice toilet brush? (2) What a dip stick…his country is floundering under his so-called leadership, Covid is rampant and he wants his face as a monument on Mount Rushmore… he's got to be kidding..right ?

*Participants’ likelihood of posting comments*

If you were to read this post on a social media site, would you comment? (Yes/No).

*Recall of comments:*

For each of the following comments, please indicate if they appeared in the comments you read.

We need to get rid of all the fucking faces on that mountain and give it back to the Lakotas and honor the treaty. Yes/No.

I can't wait to see his face up there. He's a better president than most of dead beats up there. Yes/No.

JFC- being an ass isn’t monument worthy. How about a nice toilet brush? Yes/No.

What a dip stick…his country is floundering under his so-called leadership, Covid is rampant and he wants his face as a monument on Mount Rushmore… he’s got to be kidding…right ? Yes/No.

*Engagement:*

For each of the following comments, please indicate if you would like it if you were to read it on social media. Yes / No.

*Demographic information:*

Age: ____________.

Gender: female / male / other.

Years of education: Less than high school / High school graduate / Some college / 2-year degree / 4-year degree / Professional degree/ Doctorate

Marital status: Married / Divorced / Widowed/ Separated/ Never married

Employment status: Employed full time/ Employed part-time/ Unemployed looking for work/ Unemployed not looking for work/ Retired/Student/Disabled

**Table C.1:** Demographic characteristics of the pilot study sample

Variable	Distribution
Age	*Min*_age_ = 18*, Max*_age_ = 69, *M*_age_ = 37.54
Gender	51.1% female, 47.7% male, 1.5% other
Marital status	35.3% married, 53.6% single, 8.1% divorced, 3% other
Employment status	65.9% employed, 18.1% unemployed, 5.6% retired, 7.6% student, 2.9% disabled
Education	35.6% high school level, 53.1% academic level, 10.9% professional degree, 0.4% less than high school

### Appendix D: Main Experiment

Below is the full description of the main experiment, as presented to the participants.

**Instructions**

Please read the post as well as the comments as you would normally do when reading a news article posted on Facebook. Please note that the comments were written by former participants, and your comments might be visible to future participants as well.

[Full list of articles and comments, from which a single article was presented to each participant].

**Table:**

Getting Covid 19 Twice: It is unlikely that people are getting sick with COVID-19 multiple times. Because the diagnostic tests are designed to look for parts of the virus's RNA, false positives can also arise when a dead fragment of a virus is detected in a patient- meaning that the patient made a recovery but still has bits of the virus's genetic material in his system that are not infectious	
Civil: (1) An expert virologist on the radio recently went through this. He suggested that while it may be possible to get the disease twice any second infection would usually be far less life threatening or debilitating (2) Not probable but possible… multiple medically documented cases of being hit with covid twice (3) Let's be responsible. They don't know if you can get it twice or not but, it looks likely. There is a lot that is unknown (4) I do not think you get sick again, I think for some people it lingers for a long time	Uncivil: (1) We should also be skeptical of really anything you fear mongering arseholes put out, lol (2) Thanks for the ignorant, completely uneducated perspective At least we immediately understand why college was never an option for you
Trump's face on Mount Rushmore: A White House aide asked (R) last year how an interested party might go about adding another giant presidential face to Mount Rushmore, a source told The New York Times South Dakota Gov. Kristi Noem was the first to publicly reveal Trump's desire to add his image alongside the 60-foot faces of Presidents George Washington, Thomas Jefferson, Abraham Lincoln, and Theodore Roosevelt carved into the mountain "Do you know it's my dream to have my face on Mount Rushmore?" he asked Noam, who was still in Congress at the time and running for governor. "I started laughing. He wasn't laughing, so he was totally serious," she recalled	
Civil: (1) We need to get rid of all the faces on that mountain and give it back to the Lakotas and honor the treaty (2) Such an event might not warrant a revolution, but I think we would see something like one (3) I can't wait to see his face up there. He's a better president than most of them (4) He has said he never jokes. This was not a joke	Uncivil: (1) JFC- being an ass isnt monument worthy. How about a nice toilet brush? (2) What a dip stick…his country is floundering under his so-called leadership, Covid is rampant and he wants his face as a monument on Mount Rushmore… he's got to be kidding..right ?
Canceling rent during Covid-19: The nation is facing an accelerating housing crisis. Too many people had no stable housing before the pandemic hit, and covid-19 has made the problem even worse. Renters who were already facing an affordable housing shortage now have no federal rental assistance or federal protection from eviction Lawmakers must prevent predatory investors from exploiting vulnerable families, write Elizabeth Warren Carroll Fife in Opinions	
Civil: (1) Nothing wrong with making a profit. That's the incentive to build more apartments and renting them out (2) I will be buying distressed assets. Actually, buying non performing notes is a way to help people stay in the house if they can meet adjusted conditions (3) How can these people ever pay back the back rents? (4) If you cancel rent for the renters then you must cancel mortgage payments for the owners of the rentals	Uncivil: (1) Vulture capitalists are looking to hoard housing just like they hoard money. Screw those people (2) It's a win for the dead beats who didn't pay their rent anyway. They got 6 more months free rent before being evicted
Nazi Salute Victory: Oakland A's bench coach Rayn Christenson apologized for twice performing a Nazi salute after his team's victory over Texas Rangers on Thursday night After the game, Christenson said the gesture was unintentional The moment, widely shared on social media, showed Christensons making the salute as players came in from the field at the game's conclusion. Pitcher Liam Hendriks then pushed Christenson's are into a normal "elbow bump" position. After trading a couple of elbow bumps, Christenson turned and performed the Nazi salute again,	
Civil: (1) Baseball is trying to adapt to Covid and included in that is not knowing how to even celebrate wins without high fives (2) That is just not a natural celebratory reaction (3) He wasn't even looking at the players he was supposedly trying to "karate chop." (4) I may have given him the benefit of the doubt, but the fact that he did it again immediately after being corrected shows he didn't care and knew what he was doing	Uncivil: (1) I unintentionally want to smack his face, and he should unintentionally be sacked imo… (2) Unintentional Nazi bullshit is the best kind of Nazi bullshit. What's the salary of the fool pretending it's unintentional?
Hasbro pulles Troll Dolls: A Troll doll is being pulled off store shelves amid complaints it promotes child abuse Toymaker Hasbro said Wednesday that it's in the process of removing the "trolls World Tour Giggle and Sing Poppy" from the market and will be offering customers a replacement doll of a popular female character The doll had been designed to giggle when placed in a sitting position, but some parents complain the sound activation button is inappropriately placed under the doll's skirt and between her legs An online petition suggests the doll is "conditioning" our children to think pedophilia is OK."	
Civil: (1) I think part of it is the idea of designing something "on paper." And not really visualizing how it will play out in reality (2) It's a far reach, but it's also very poor toy design. That is all (3) Good glad to hear they taking them off the shelves but this toy shouldn't of been made that way in first place (4) Hard to believe this made it to market	Uncivil: (1) How did this get past the think tank? PERVERTED BS (2) Not to mention, which parent would buy this? Fucking sick people, regardless which side

*Participants’ comment:*

Add your own comment…

*Demographic information:*

Age: ____________.

Gender: female / male / other.

## Footnotes

1 https://blog.firstreference.com/website-post-comments-can-be-liable-for-defamation/#.X5U0u9Aza00.

2 We note that other factors, such as rationality (Schäfer et al., 2022), can have a similar effect making people feel uncomfortable to express their views.

3 Habermas’s (1989) normative ideals of deliberative democracy in the “public sphere” are referenced in almost all works discussing freedom of expression online. However, it is important to acknowledge that other, later theories of democracy (e.g., liberal individualist; communitarian) are less demanding regarding the civility of contributions to the discussion (see Freelon, 2010 for a review of models of democratic communication in the context of online expression).

4 According to Krippendorff (2004), an inter-coder reliability coefficient of 0.667 is suitable for drawing tentative conclusions. However, studies that classify subjective data similar to online incivility report relatively lower levels of agreement. For example, Mulki et al. (2019) reported an agreement level of 0.64 in their study on hate speech on Twitter. Similarly, Moon et al. (2020) found an agreement level of 0.492 and 0.496 in their study of toxic comments in Korean entertainment news, while Chakravarthi (2020) reported an agreement level of 0.63 in their study of hope speech for equality, diversity, and inclusion in social media comments.

5 We chose Facebook as the design for the controlled lab experiment due to its popularity. We wanted users to feel it’s a familiar website that they know and use often. However, we also had to utilize a design that allows for clear serial positioning, and the six comments allowed us to have clear beginning, middle and an end.

6 We added these two additional uncivil comments to disguise the purpose of the study, and we did not analyze participants’ responses with regard to these comments.

7 We note that it is possible that in practice, the serial position of comments might result in a self-selection bias, implying that the type of commenters changes given the environment, and, in particular, the number of comments that have been already posted. We could not examine this effect in our experiments.

8 Ten participants were excluded from the analyses because, when asked to comment on the article, they wrote that they would not comment.
